# 63230 Quantifying Heavy Metals in Interstitial Fluid for Remote Monitoring of Chronic Exposures

**DOI:** 10.1017/cts.2021.758

**Published:** 2021-03-30

**Authors:** Robert Taylor, Alicia Bolt, Abdulmehdi Ali, Yiliang Zhu, Justin Baca

**Affiliations:** 1 University of New Mexico Health Sciences Center; 2 University of New Mexico

## Abstract

ABSTRACT IMPACT: We present a minimally-invasive approach to monitoring heavy metal exposures in geographically disperse populations; this framework may encourage and facilitate greater participation in a broad range of related clinical studies that require biosampling. OBJECTIVES/GOALS: We hypothesize that microneedle array (MA) extraction of interstitial fluid (ISF) will enable minimally-invasive quantitation of heavy metal (HM) exposure. We establish analytical parameters for ICP-MS analysis of HMs in ISF, quantify baseline HM content in ISF vs other fluids, and characterize a mixed HM exposure model. METHODS/STUDY POPULATION: Recent advances in ISF extraction and analysis suggest a minimally-invasive to monitor HM exposure longitudinally in both urban and dispersed communities. ISF can be collected with MAs and is a rich source of disease and exposure biomarkers. To refine analytical methods, Human subjects with no underlying skin disease were recruited into the IRB-approved study. Each subject had blood and urine collected. ISF was also collected using 3D-printed MA-holders. The fluids were analyzed, using ICP-MS, to quantify the levels of uranium (U), cadmium (Cd), vanadium (V), and arsenic (As). Additionally, we analyzed 2,770 subjects from the public NHANES dataset from 2018-2019. Python and Scikit-learn were used to analyze the demographics, survey responses, and metal concentrations for these individuals. RESULTS/ANTICIPATED RESULTS: While several studies have described the surface water and sediment content of toxic metals, determining biological loads remains challenging due to the need to collect blood or urine from a dispersed rural population over time. Our preliminary results suggest similar HM concentrations in ISF, compared with blood in a small unexposed population. Analysis of subjects from the NHANES public datasets suggest similar baseline blood HM concentrations in diverse subject populations with some differences in Cd depending on smoking and e-cigarette usage. Correlation maps also suggest possible synergy between different metals with cobalt and chromium showing the highest correlation. The initial results from this study have been applied to develop a mixed HM exposure model in rats for further translational testing. DISCUSSION/SIGNIFICANCE OF FINDINGS: We present a minimally-invasive HM monitoring approach. Exposure to multiple HMs is suspected to have additive or synergistic harmful health effects. We ultimately envision a wearable microneedle patch that could be mailed to individuals or distributed through community centers, worn for a few hours, and returned to a central laboratory.

